# Influence of Rural and Urban Environments on Lifestyle, Dietary Patterns, and Oral Health Among Adolescents in Mallorca: A Cross-Sectional Study

**DOI:** 10.3390/children13050645

**Published:** 2026-05-04

**Authors:** Irene Coll Campayo, Pablo Estebala Alández, Daniela Vallejos Rojas, Raúl Cuesta Román, María Luisa Bonet, Nora López-Safont

**Affiliations:** 1 Faculty of Dentistry, University ADEMA University School, C. Passamaners 11, 07009 Palma, Spain; i.coll@eua.edu.es (I.C.C.); p.estebala@eua.edu.es (P.E.A.); d.vallejos@eua.edu.es (D.V.R.); r.cuesta@eua.edu.es (R.C.R.); 2 ADEMA-Health Group of University Institute for Research in Health Sciences (IUNICS), 07122 Palma, Spain; 3 Nutrigenomics, Biomarkers and Risk Evaluation (NuBE) Research Group, University of the Balearic Islands, 07122 Palma, Spain; luisabonet@uib.es; 4 CIBER Fisiopatología de la Obesidad y Nutrición (CIBEROBN), Instituto de Salud Carlos III (ISCIII), 28029 Madrid, Spain; 5 Artificial Intelligence Research Institute of the Balearic Islands (IAIB), 07122 Palma, Spain; 6 Institut d’Investigació Sanitària Illes Balears (IdISBa), 07120 Palma, Spain; 7 Biology Department, University of Balearic Islands, Ctra. Valldemossa Km 7.5, 07122 Palma, Spain

**Keywords:** adolescents, dietary patterns, oral health, rural health, urban health, ultra-processed foods, DMFT, lifestyle, public health

## Abstract

**Highlights:**

**What are the main findings?**

- Adolescents living in urban areas showed higher consumption of sugar-sweetened beverages and ultra-processed foods.
- Adolescents living in rural areas had higher levels of physical activity but a greater prevalence of dental caries.
- Oral hygiene behaviors were similar between rural and urban adolescents, despite differences in oral health outcomes.
- Periodontal health indicators were slightly better among rural adolescents compared to urban peers.

**What are the implications of the main findings?**

- Public health strategies should address unhealthy dietary patterns in urban adolescents, particularly reducing ultra-processed food consumption.
- Improving access to preventive dental services in rural areas is essential to reduce caries prevalence.
- Interventions should consider that oral health disparities are not fully explained by individual behaviors, highlighting the role of structural determinants.
- Health promotion programs should be adapted to geographic context to effectively reduce health inequalities among adolescents.

**Abstract:**

Background/Objectives: Adolescence is a critical developmental stage during which lifestyle and dietary habits are established, influencing both general and oral health outcomes. Territorial disparities between rural and urban environments may contribute to nutritional inequalities and health vulnerabilities. The aim of this study was to analyze differences between rural and urban environments in terms of lifestyle behaviors, dietary patterns, and oral health outcomes among adolescents in Mallorca, Spain. Methods: A cross-sectional study was conducted among 463 adolescents corresponding to the WHO index ages (12 and 15 years). Data were collected through questionnaires assessing dietary habits, oral hygiene behaviors and lifestyle characteristics. Clinical oral examinations were performed following the World Health Organization Pathfinder methodology. Statistical analyses included descriptive statistics and comparative analyses between rural and urban populations. Results: Urban adolescents reported higher consumption of sugar-sweetened beverages and ultra-processed foods (*p* < 0.001), whereas rural adolescents showed higher weekly physical activity (4.45 ± 2.34 vs. 3.62 ± 2.41 h/week; *p* < 0.001). Caries prevalence was higher in rural students (45.0% vs. 28.6%; *p* < 0.001), who however demonstrated better periodontal indicators. Conclusions: Geographic environment is associated with differences in dietary patterns, physical activity levels, lifestyle behaviors, and oral health outcomes among adolescents. These findings highlight the importance of targeted public health interventions adapted to geographic context and support the role of broader social and environmental determinants in adolescent oral health.

## 1. Introduction

Social and environmental determinants play a decisive role in shaping lifestyle behaviors and contributing to health inequalities from early life stages. Factors such as socioeconomic status, educational environment, availability of community resources, and type of residence—urban or rural—directly influence diet, physical activity, and both general and oral health outcomes in children and adolescents [1,2]. In this context, international organizations such as the World Health Organization (WHO) and the European Commission have emphasized the importance of addressing territorial and social inequalities in child health as a public health priority [3,4,5].

Adolescence represents a critical developmental stage for the consolidation of lifestyle behaviors that may persist into adulthood. During this period, individuals progressively gain autonomy in dietary choices and physical activity patterns, although these behaviours remain strongly influenced by family, school, and broader socioeconomic environments [6,7]. Evidence suggests that diet quality and the adoption of healthy lifestyles adoption during adolescence are associated with parental education level and socioeconomic status, which are commonly used as proxy indicators of social position [8,9]. Therefore, adolescence constitutes a key window for the establishment of long-term behavioral patterns with potential health consequences [10].

Physical activity is an essential component of adolescent health and is widely associated with overall well-being and lifestyle patterns [11]. Although its direct relationship with oral health is not fully established, it is considered part of a broader cluster of health-related behaviors [12]. Adolescents who are more physically active tend to exhibit healthier dietary patterns and greater awareness, which may indirectly contribute to better oral health outcomes [13].

Urban and rural environments provide distinct structural and social conditions that influence access to food marketing and higher commercial availability contributes to increased consumption of ultra-processed foods, sugar-sweetened beverages, and fast food products [14,15,16]. In contrast, rural environments may present barriers to accessing preventive healthcare services including dental care [6,7,8], and are sometimes associated with reduced opportunities for active transportation and recreational physical activity [7,17,18]. These structural inequalities may contribute to long-term differences in both general and oral health outcomes in pediatric populations [5,9].

Recent evidence from school-based populations in Mallorca has shown that geographic environment (urban or rural), type of school and parental education level significantly influence dietary behaviours and oral status. In particular, higher consumption of sugar rich products and differences in caries prevalence have been observed according to socioeconomic and territorial factors [17]. However, most available studies focus on younger children, while evidence regarding rural–urban differences during adolescence remains limited [5]. This is particularly relevant, as adolescence is a stage in which health behaviours become increasingly independent from parental control.

In light of this evidence, the primary aim of the present study was to analyze differences in lifestyle behaviors, dietary patterns, and oral health outcomes among adolescents living in rural and urban areas of Mallorca.

As a secondary objective, the study explored the relationship between these lifestyle and dietary factors and oral health indicators, in order to better understand potential determinants of dental caries and periodontal status in this population.

## 2. Materials and Methods

### 2.1. Study Design

An observational cross-sectional epidemiological study was conducted in a school-based population in Mallorca (Spain) to analyze differences in lifestyle behaviours and oral health status between adolescents living in rural and urban environments. The study was designed according to the World Health Organization (WHO) recommendations for oral health surveys, applying the Pathfinder methodology [19]. This approach is based on a stratified cluster sampling design that includes key population subgroups with potentially different levels of disease, in order to improve representativeness [19,20].

The source of information for the sampling sites (schools) was obtained from official registries of the General Directorate of Planning, Management, and Centers of the Autonomous Community of the Balearic Islands (CAIB) and the National Institute of Statistics (INE). The target population included schoolchildren corresponding to the WHO index ages of 12 and 15 years [19]. The sample size was calculated in the following way: for a population of 12,000 children, and a caries prevalence proportion of 0.35, a minimum sample size of approximately 340 children was required to attain a 95% confidence level with a 5% margin of error. However, we opted to increase the sample size to enhance the accuracy of our effect estimates, reduce the margin of error, and improve the statistical power of the study. A total of 463 adolescents (12–15 years) were analyzed—specifically, 230 adolescents aged 12 years (girls *n* = 105, boys *n* = 125) and 233 adolescents aged 15 years (girls *n* = 121, boys *n* = 112). An effort was made to maintain a balanced distribution by sex across cohorts.

Stratification criteria included geographic location (urban vs. rural), type of school (public vs. charter/private), and age group (12 and 15 years). After stratification, schools were selected using systematic random sampling, applying proportional allocation to ensure representativeness of each stratum according to the characteristics of the study area.

Within each selected school, participants were recruited from the corresponding age groups. All eligible students were invited to participate. Prior to data collection, detailed information about the study was provided to parents or legal guardians, and written informed consent was obtained. Only students with signed consent were included in the study.

The primary outcome of the study was the comparison of lifestyle behaviors, dietary patterns, and oral health indicators between adolescents living in rural and urban environments. Secondary outcomes included the exploration of the relationship between lifestyle and dietary factors and oral health variables.

The study protocol was approved by the Research Ethics Committee of the Balearic Islands (CEI IB3737/18) and complied with current legislation, the Declaration of Helsinki, and good clinical practice standards.

### 2.2. Data Collection and Study Variables

Sociodemographic, oral health, lifestyle, and dietary variables were collected between November 2018 and December 2019.

Oral health data were obtained using the standardized forms from the World Health Organization (WHO) Oral Health Surveys: Basic Methods [19], specifically the clinical assessment form (Annex 2) and the oral health questionnaire for children (Annex 8). Clinical examinations were performed under standardized lighting conditions using a portable headlamp, No. 5 dental mirrors, WHO periodontal probes, and standardized participant positioning.

Prior to data collection, examiners underwent a structured training and calibration process following the World Health Organization (WHO) guidelines [19]. This included theoretical and practical sessions, as well as duplicate clinical examinations to assess inter-examiner reliability. Agreement was evaluated using the Kappa statistic, obtaining a mean value of 0.757, indicating substantial agreement. A reference examiner (gold standard) was also used to ensure consistency across examiners. A more detailed description of the calibration procedure has been previously reported elsewhere [21,22].

Lifestyle variables, were assessed using a physical activity questionnaire based on the International Physical Activity Questionnaire (IPAQ), validated and used in Spanish studies like the ENALIA study [23,24].

Dietary habits were assessed through questionnaires designed to capture nutrition-related knowledge, attitudes, and practices (KAP), adapted from the Food and Agriculture Organization (FAO) guidelines, Guidelines for Assessing Nutrition-Related Knowledge, Attitudes and Practices (FAO, 2014) [25]. In addition, a food consumption frequency questionnaire was administered following the methodology proposed by the European Food Safety Authority (EFSA), as outlined in the EU Menu project and the document General Principles for the Collection of National Food Consumption Data in the View of a Pan-European Dietary Survey (EFSA, 2009) [26]. The foods and beverages analyzed were classified according to the NOVA food classification system, which categorizes foods based on the extent and purpose of industrial processing into four groups: (1) unprocessed or minimally processed foods, (2) processed culinary ingredients, (3) processed foods, (4) ultra-processed foods and beverages [27]. Food consumption frequency was originally collected using seven response categories: “never,” “1–3 times per month,” “once per week,” “2–3 times per week,” “4–6 times per week,” “once per day,” and “more than once per day.” For analytical purposes, these categories were grouped into three broader, mutually exclusive categories: low frequency (≤1 time/week), moderate frequency (2–6 times/week), and high frequency (≥1 time/day).

In addition, information on maternal educational level was collected as an indicator of socioeconomic background. This variable was obtained using the questionnaire from the (WHO) Oral Health Surveys: Basic Methods [19]. Educational level was categorized according to the highest level of education attained and was used to reflect socioeconomic and educational diversity within the study population.

### 2.3. Variables Analyzed

The variables analyzed were categorized as follows:

- Sociodemographic variables: geographic location of the school (rural or urban), maternal education level (primary, secondary, higher education).
- Oral health variables: Community Periodontal Index (number of healthy sextants, gingival bleeding, or dental calculus) and DMFT index (decayed, missing, and filled teeth), tooth brushing frequency, dental floss use, toothpaste use, dental pain frequency, gingival pain frequency and self-perceived dental and gingival health status.
- Lifestyle variables: number of weekly hours of sports practice and type of school commute (walking, bicycle, car, or public transport).
- Dietary variables: frequency of consumption of different food and beverage groups, classified according to the NOVA food processing classification system. Consumption frequency was categorized into low (≤1 time/week), moderate (2–6 times/week), and high (≥1 time/day).

### 2.4. Statistical Analysis

Data were analyzed using IBM SPSS Statistics software version 27.0.1.0 (IBM Corp., Armonk, NY, USA). Student’s *t*-tests were applied for comparisons of means, and chi-square (χ^2^) tests were used to compare proportions between rural and urban groups. Statistical significance was established at *p* < 0.05.

The analysis was primarily exploratory and based on bivariate comparisons to assess differences according to geographic location. No multivariable adjusted models were performed.

The analysis was conducted following the Strengthening the Reporting of Observational Studies in Epidemiology (STROBE) recommendations for cross-sectional studies [28].

## 3. Results

The total sample consisted of 463 adolescents aged 12 (sixth-year elementary) and 15 years (fourth-year secondary), distributed evenly across both age groups and geographic environments. Regarding geographic location, 47.94% (*n* = 222) corresponded to students from rural areas and 52.05% (*n* = 241) to students from urban areas (Table 1).

### 3.1. Periodontal Status

Table 2 shows the distribution of maximum Community Periodontal Index (CPI) codes according to geographic environment. The overall distribution of CPI categories differed significantly between rural and urban adolescents (*p* = 0.039).

A higher proportion of rural participants presented a healthy periodontium (29.7% vs. 22.8%), whereas bleeding on probing and calculus was more frequent among urban adolescents (28.2% vs. 24.8% and 36.1% vs. 31.1%, respectively).

### 3.2. Dental Caries Experience

A significant association was observed between geographic location and caries experience. Students living in urban environments showed better caries health status compared with those in rural environments. Caries prevalence was 45% in the rural group and 28.6% in the urban group (*p* < 0.001). Similarly, the DMFT index was significantly higher in the rural environment (1.11 ± 1.69) than in the urban environment (0.56 ± 1.12) (*p* < 0.001) (Table 3).

### 3.3. Education Level and Oral Health

A statistically significant association was observed between parental educational level and caries prevalence (*p* = 0.019). Adolescents whose tutors had lower educational levels showed higher prevalence of dental caries, with 25.0% in the primary education group compared to 7.5% in those with higher education.

No statistically significant differences were observed in DMFT values according to parental educational level (*p* = 0.096), although a decreasing trend was noted with increasing educational attainment (Table 4).

### 3.4. Mode of Transportation to School

Mode of transportation to school differed significantly according to geographic location (*p* = 0.001). The use of motorized transport (bus/car) was similar in rural and urban areas (54.1% vs. 52.9%). Walking was more frequent in urban environments (42.9%) compared with rural settings (31.9%), whereas bicycle use was higher in rural areas (11.1% vs. 3.3%). The use of other modes of transportation was low in both groups (Table 5).

### 3.5. Weekly Hours of Physical Activity

Geographic location significantly influenced the weekly hours of sports participation. Students from rural areas reported 23.2% more hours of physical activity compared with those from urban areas (rural: 4.45 ± 2.34 h/week; urban: 3.62 ± 2.41 h/week; *p* < 0.001) (Table 6).

### 3.6. Frequency of Food Consumption

Significant differences were observed between urban and rural students regarding the frequency of consumption of several food and beverage categories, specifically the following: coffee (*p* < 0.001), diet soft drinks (*p* < 0.001), sugar-sweetened soft drinks (*p* < 0.001), energy drinks (*p* < 0.001), isotonic beverages (*p* < 0.001), industrial fruit juices (*p* < 0.001), packaged milkshakes (*p* < 0.001), oily fish (*p* < 0.001), processed meats (*p* = 0.039), hamburgers (*p* < 0.001), legumes (*p* = 0.020), nuts (*p* = 0.020), salty snacks (*p* < 0.001), potato chips (*p* < 0.001), and whole-grain bread (*p* = 0.040) (Table 7).

Overall, adolescents from urban environments showed a higher proportion of high-frequency consumption (≥1 time/day) of ultra-processed foods and sugar-sweetened beverages compared with those from rural environments, who more frequently fell into the low-frequency consumption category (≤1 time/week).

### 3.7. Oral Hygiene Behaviours and Self-Perceived Oral Health

Overall, oral hygiene behaviors and self-perceived oral health were similar between rural and urban adolescents. No statistically significant differences were observed in tooth brushing frequency, dental floss use, toothpaste use (with the exception noted below), self-perceived dental or gingival health, or reported dental and gingival pain (all *p* > 0.05).

A statistically significant difference was observed in toothpaste use, which was higher among urban adolescents (*p* = 0.046). In addition, a non-significant trend toward higher gingival pain frequency was observed among urban participants (*p* = 0.060) (Table 8).

## 4. Discussion

The findings of this study indicate that geographic environment is associated with lifestyle behaviors, dietary patterns, and oral health outcomes among adolescents in Mallorca. The impact of social, economic, and environmental context on child and adolescent health has been widely recognized, as the conditions in which young people live may shape health-related behaviors and overall well-being [29].

From an oral health perspective, dental caries remains one of the most prevalent non-communicable diseases among children and adolescents and is strongly associated with unhealthy lifestyles, particularly frequent consumption of free sugars and inadequate control of dental biofilm [9,30]. Evidence suggests that the frequency and pattern of sugar intake may have a greater impact on caries risk than total sugar quantity consumed [31]. Conversely, diets rich in fruits, vegetables, and dairy products have been associated with lower caries risk due to their content of fiber, polyphenols, and calcium, as well as their capacity to stimulate salivary flow [32,33].

In the present study, adolescents from urban environments exhibited a less healthy dietary pattern, characterized by a higher consumption of ultra-processed foods and sugar-sweetened beverages. These findings are consistent with the HELENA study, which demonstrated an association between urbanization, greater access to industrialized foods, and lower adherence to the Mediterranean diet [34]. Other studies have shown that the consumption of ultra-processed foods is associated with increased risks of obesity, metabolic disorders, and deterioration of oral health [6,35].

Despite reporting lower consumption of ultra-processed foods and sugar-sweetened beverages and higher levels of physical activity, students from rural environments presented a higher prevalence of dental caries and a higher DMFT index compared with their urban counterparts. These results are consistent with previous studies conducted in similar populations [17] and with research indicating that rural environments have been associated with higher caries incidence. However, these differences may reflect broader contextual and structural factors not directly assessed in the present study, rather than specific individual-level behaviors.

However, rural students demonstrated better periodontal indicators, with lower gingival bleeding and a higher proportion of healthy periodontium. These differences may be related to variations in unmeasured behavioral or contextual factors, such as dietary patterns or oral hygiene practices [17,32].

Regarding oral hygiene behaviors, no significant differences were observed between rural and urban adolescents, except for a slightly higher use of toothpaste in urban participants. Self-perceived oral health and reported dental and gingival pain were also similar between groups. These findings suggest that differences in oral health outcomes, particularly caries prevalence, are unlikely to be explained by individual hygiene practices alone and support the possibility that broader contextual and socioeconomic determinants may contribute. This interpretation is consistent with recent evidence indicating that dental caries is a multifactorial condition influenced not only by behavioral factors such as oral hygiene, but also by socioeconomic status, environmental context, and access to resources [36]. Furthermore, studies in adolescent populations have shown that socioeconomic and psychosocial factors play a significant role in oral health outcomes, including self-perceived oral health and periodontal status [37,38].

In the present study, parental educational level was significantly associated with caries prevalence, with higher prevalence observed among adolescents whose tutors had lower educational attainment. This finding is consistent with previous literature identifying socioeconomic status as a key determinant of oral health, influencing both health behaviors and access to preventive care [36,37,38]. Adolescents from rural environments reported a greater number of weekly hours of physical activity, suggesting that environmental factors such as greater availability of open spaces may facilitate more active lifestyles. Although physical activity is generally associated with healthier behavioral patterns, including improved dietary habits and greater health awareness, no direct association with oral health outcomes was observed in this study. This finding reinforces the multifactorial nature of dental caries, indicating that a range of behavioral, socioeconomic, and contextual factors may contribute to oral health outcomes [39].

These findings are consistent with previous studies conducted in similar adolescent populations, which have also reported a higher burden of dental caries in rural settings despite more favorable behavioral profiles in urban areas [22]. Similar patterns have been described in studies from other European regions, where rural residence has been associated with increased caries experience, potentially reflecting broader structural and socioeconomic inequalities rather than individual behavioral differences alone [40]. In contrast, some studies have reported no significant differences in caries prevalence between rural and urban adolescents after adjustment for socioeconomic status and access to care, highlighting the heterogeneity of findings across populations [41].

More broadly, multilevel evidence suggests that adolescent oral health is strongly influenced by socioeconomic and contextual determinants, including parental education, healthcare accessibility, and broader structural inequalities [36].

Connected to the physical activity results, another relevant finding was the difference in school commuting patterns. Mode of transport to school varied according to geographic environment. While bicycle use was more frequent among rural adolescents, walking was more common in urban environments, and the use of motorized transport was similar in both groups. This pattern is consistent with previous research demonstrating that active transport is associated with increased daily physical activity and improved cardiovascular health outcomes in children [42,43].

Overall, the results reinforce the concept that geographic environment may act as a social determinant of health [44,45,46], influencing both behavioral habits and oral health outcomes. The observed differences between rural and urban adolescents highlight the need for tailored health promotion strategies. In rural environments, interventions should prioritize improving access to preventive dental services and strengthening oral health education, whereas in urban settings, strategies should focus on reducing consumption of ultra-processed foods and promoting regular physical activity.

Finally, several limitations should be considered. The cross-sectional design does not allow causal relationships to be established, and self-reported information may be subject to recall bias or social desirability bias. In addition, the statistical analysis was based on bivariate comparisons and did not include multivariable models to control for potential confounding factors such as age, sex, or school type. Therefore, the observed associations should be interpreted with caution. Nevertheless, the use of standardized WHO Pathfinder methodology [19] and validated instruments strengthens the reliability of the findings.

Future longitudinal studies are needed to better understand causal pathways linking geographic environment, lifestyle behaviors, and oral health outcomes during adolescence, as well as to evaluate the effectiveness of targeted preventive interventions adapted to different territorial contexts.

## 5. Conclusions

In conclusion, adolescents from rural and urban environments in Mallorca showed significant differences in lifestyle behaviours, dietary patterns, and oral health outcomes. These findings suggest the influence of geographic context as an important determinant of both general and oral health during adolescence.

Rural adolescents reported higher levels of physical activity and more active transport to school; however, no direct association with oral health outcomes was observed, reinforcing the multifactorial nature of dental caries.

Additionally, oral hygiene behaviors were similar between groups, suggesting that the observed differences in caries prevalence may be more strongly associated with structural and contextual factors than to individual hygiene practices.

The results suggest the need for targeted public health policies aimed at reducing territorial inequalities and promoting healthier environments for children and adolescents. Addressing geographic disparities from early life represents a strategic opportunity to improve both oral and overall health outcomes in the Balearic population.

## Figures and Tables

**Table 1:** Sample distribution by sex, school type, and geographic location.

		12 Years		15 Years		Total	
		*n*	%	*n*	%	*n*	%
Sex	Male	125	54.34	112	48.06	237	51.18
	Female	105	45.65	121	51.93	226	48.81
School Type	Public	159	69.13	190	81.54	349	75.37
	Subsidized/private	71	30.86	43	18.45	114	24.62
Geographic location	Urban	140	60.86	101	43.34	241	52.05
	Rural	90	39.13	132	56.65	222	47.94

(*n*, sample size).

**Table 2:** Community Periodontal Index (CPI) by Geographic Location.

Geographic Location	*N*	CPI 0 Healthy *n* (%)	CPI 1 Bleeding *n* (%)	CPI 2 Calculus *n* (%)	*p*-Value
Rural	190	66 (29.7%)	55 (24.8%)	69 (31.1%)	0.039 *
Urban	210	55 (22.8%)	68 (28.2%)	87 (36.1%)	

(*n*, sample size; chi-square: * variable with significant effect (*p* < 0.05)).

**Table 3:** Dental Caries Prevalence and Experience by Geographic Location.

Geographic Location	*N*	Caries Prevalence (%)	*p*-Value (Chi Square)	Active Caries Lesions *n* (%)	*p*-Value (Chi-Square)	DMFT Index Mean ± SD	*p*-Value
Rural	222	100 (45%)	<0.001	33 (14.8%)	0.058	1.11 ± 1.69	<0.001 *
Urban	241	69 (28.6%)		22 (9.12%)		0.56 ± 1.12	

(*n*, sample size; Chi-square test for proportions; Student’s *t*-test for DMFT: * variable with significant effect (*p* < 0.001)).

**Table 4:** Association between maternal educational level, caries prevalence, and geographic location.

Parental Education Level	*n*	Caries Prevalence (%)	*p*-Value	DMFT (Mean ± SD)	*p*-Value
Primary	20	25.0%	0.019 *	1.10 ± 1.48	0.096
Secondary	95	13.7%		1.06 ± 1.49	
Higher education	228	7.5%		0.71 ± 1.42	

(*n*, sample size; Chi-square test for caries prevalence; Student’s *t*-test for DMFT; * variable with significant effect (*p* < 0.05)).

**Table 5:** Mode of Transportation to School According to Geographic Location.

Geographic Location	*N*	Bus/Car	Bicycle	Walking	Other	*p*-Value
Rural	216	117 (54.1%)	24 (11.1%)	69 (31.9)	6 (2.77)	0.001 *
Urban	240	127 (52.9%)	8 (3.33%)	103 (42.9)	2 (0.83%)	

(*n*, sample size; chi-square test, * variable with significant effect (*p* < 0.05)).

**Table 6:** Weekly Hours of Physical Activity According to Geographic Locations.

Variable	Geographic Locations	*n*	Mean (Hours/Week)	SD	SE	95% CI	*p*-Value
Weekly hours of physical activity	Rural	176	4.45	2.34	0.17	(4.10–4.80)	<0.001 *
	Urban	193	3.61	2.41	0.17	(3.27–3.95)	

(*n*, sample size; SD, standard deviation; SE, standard error; CI, confidence interval; Student’s *t*-test, * variable with significant effect (*p* < 0.05)).

**Table 7:** Frequency of Food Consumption among Students According to Geographic Location.

Food Category	Consumption Frequency	Total *n*	%	Rural n	Urban n	*p*-Value
Coffee	Low	331	75.2%	182	149	<0.001 *
	Moderate	14	3.2%	10	4	
	High	95	21.6%	19	76	
Diet soft drinks	Low	329	73.1%	184	145	<0.001 *
	Moderate	50	11.1%	26	24	
	High	71	15.8%	8	63	
Sugar-sweetened soft drinks	Low	278	61.6%	153	125	<0.001 *
	Moderate	90	20.0%	43	47	
	High	83	18.4%	22	61	
Energy Drinks	Low	359	79.4%	199	160	<0.001
	Moderate	21	4.6%	12	9	
	High	72	15.9%	7	65	
Isotonic beverages/Sports drinks	Low	311	69.3%	170	141	<0.001 *
	Moderate	70	15.6%	35	35	
	High	68	15.1%	12	56	
Industrial fruit juice	Low	246	55.5%	136	110	<0.001 *
	Moderate	93	21.0%	44	49	
	High	104	23.5%	32	72	
Packaged milkshakes	Low	304	67.7%	173	131	<0.001 *
	Moderate	62	13.8%	25	37	
	High	83	18.5%	18	65	
Oily Fish	Low	346	76.0%	195	151	<0.001 *
	Moderate	62	13.6%	21	41	
	High	47	10.3%	3	44	
Processed meats/cold cuts	Low	130	28.8%	52	78	0.039 *
	Moderate	191	42.3%	93	98	
	High	131	29.0%	73	58	
Hamburgers	Low	330	73.3%	175	155	<0.001 *
	Moderate	67	14.9%	32	35	
	High	53	11.8%	9	44	
Legumes	Low	209	46.1%	114	95	0.020 *
	Moderate	194	42.8%	85	109	
	High	50	11.0%	18	32	
Nuts	Low	259	57.0%	134	125	0.020 *
	Moderate	135	29.7%	65	70	
	High	60	13.2%	19	41	
Salty Snacks	Low	310	68.3%	169	141	<0.001 *
	Moderate	90	19.8%	38	52	
	High	54	11.9%	12	42	
Potato chips	Low	306	67.3%	168	138	<0.001 *
	Moderate	93	20.4%	36	57	
	High	56	12.3%	15	41	
Whole-grain bread	Low	244	54.3%	133	111	0.004 *
	Moderate	98	21.8%	44	54	
	High	107	23.8%	38	69	

(*n*: sample size; chi-square: * variable with significant effect (*p* < 0.05)).

**Table 8:** Oral hygiene behaviors and self-perceived oral health according to geographic location.

Variable	Total *n*	Rural (Mean ± SD)	Urban (Mean ± SD)	*p*-Value
Tooth brushing frequency	460	5.21 ± 1.33	5.34 ± 1.27	0.144
Dental floss use	349	1.74 ± 0.43	1.74 ± 0.43	0.487
Toothpaste use	428	1.00 ± 0.09	1.12 ± 0.92	0.046
Self-perceived dental health	435	3.01 ± 1.01	2.99 ± 0.99	0.405
Self-perceived gingival health	453	3.43 ± 1.58	3.59 ± 1.77	0.165
Dental pain frequency	455	3.54 ± 1.33	3.54 ± 1.28	0.491
Gingival pain frequency	446	3.65 ± 1.38	3.87 ± 1.56	0.060

(*n*, sample size; SD, standard deviation; Student’s *t*-test).

## Data Availability

The datasets generated and/or analyzed during the current study are not publicly available as they are being utilized for ongoing purposes, but they are available from the corresponding author on reasonable request.

## Abbreviations

The following abbreviations are used in this manuscript:

CPI	Community Periodontal Index
DMFT	Decayed, Missing, and Filled Teeth
WHO	World Health Organization
SD	Standard Deviation
SE	Standard Error
CI	Confidence Interval
NOVA	Food Processing Classification System
CAIB	Autonomous Community of the Balearic Islands
INE	National Institute of Statistics
